# Regulation of S-formylglutathione hydrolase by the anti-aging gene klotho

**DOI:** 10.18632/oncotarget.19111

**Published:** 2017-07-08

**Authors:** Yuechi Xu, Zhongjie Sun

**Affiliations:** ^1^ Department of Physiology, College of Medicine, University of Oklahoma Health Sciences Center, Oklahoma City, OK, USA

**Keywords:** klotho, receptor, binding, S-formylglutathione hydrolase, GSH, Gerotarget

## Abstract

Klotho is an aging-suppressor gene. The purpose of this study is to investigate the binding sites (receptors) and function of short-form Klotho (Skl). We showed that Skl physically bound to multiple proteins. We found physical and functional interactions between Skl and *S*-formylglutathione hydrolase (FGH), a key enzyme in the generation of the major cellular anti-oxidant GSH, using co-immunoprecipitation-coupled mass spectrometry. We further confirmed the colocalization of Skl and FGH around the nucleus in kidney cells using immunofluorescent staining. Skl positively regulated FGH gene expression *via* Kid3 transcription factor. Overexpression of Skl increased FGH mRNA and protein expression while silencing of Skl attenuated FGH mRNA and protein expression. Klotho gene mutation suppressed FGH expression in red blood cells and kidneys resulting in anemia and kidney damage in mice. Overexpression of Skl increased total GSH production and the GSH/GSSG ratio, an index of anti-oxidant capacity, leading to a decrease in intracellular H_2_O_2_ and superoxide levels. The antioxidant activity of Skl was eliminated by silencing of FGH, indicating that Skl increased GSH *via* FGH. Interestingly, Skl directly interacted with FGH and regulated its function. Site-directed mutagenesis of the *N*-glycan-modified residues in Skl abolished its antioxidant activity, suggesting that these *N-*glycan moieties are important features that interact with FGH. Specific mutation of Asp to Ala at site 285 resulted in a loss of anti-oxidant activity of Skl, suggesting that *N*-glycosylation at site 285 is the key mechanism that determines Skl activity. Therefore, this study demonstrates, for the first time, that Skl regulates anti-oxidant GSH generation *via* interaction with FGH through *N*-glycosylation.

## INTRODUCTION

Klotho was originally discovered as an aging-suppressor gene [1]. Mutation of the Klotho gene (*KL*) leads to numerous premature-aging phenotypes, including soft tissue calcification, emphysema, sex gland dysplasia, infertility, and hyperphosphatemia [1]. Overexpression of Klotho slows down the aging process and extends lifespan in mice [2]. Klotho is predominantly expressed in the distal tubules of the kidneys and the choroid plexus of the brain [1, 3]. Two forms of Klotho have been identified: full-length Klotho (Fkl) and short-form Klotho (Skl). These two forms are generated by alternative RNA splicing of the *KL* gene. Fkl is a single transmembrane protein and contains two extracellular domains, KL1 and KL2, belonging to the glycosyl hydrolase family [4]. In addition, Fkl shows the *N*-glycosylation post-translational modification. Different glycosylated Fkl proteins exhibit different apparent mobilities in SDS-PAGE [5]. Fkl forms a complex with FGFR1c, FGFR3c, or FGFR4 to enhance the affinity of FGF23 to this complex on the cell membrane [6]. The association between FGF23 and its receptors activates the mitogen-activated protein kinase (MAPK) cascade, inhibits phosphate reabsorption in kidney proximal tubule cells, and suppresses expression of sodium-dependent phosphate co-transporter type 2c, which mediates phosphate reabsorption. In addition, Klotho deficiency increases oxidative stress, contributing to aging phenotypes [7-10].

By contrast, the function and intracellular localization of Skl are unclear. The binding partners of Skl are important for understanding its function but have never been investigated. Skl contains only one glycosyl hydrolase domain and does not contain a transmembrane domain [11]. The purpose of this study is to identify the binding proteins of Skl and investigate their functional interactions. One interesting finding is that Skl binds to *S*-formylglutathione hydrolase (FGH).

FGH, a glutathione thiol esterase expressed by the esterase D gene, catalyzes the conversion of *S*-formylglutathione to glutathione (GSH), the major anti-oxidant in the cell, and formate [12-16]. FGH has been characterized in humans [13], *Arabidopsis thaliana* [17, 18], *Saccharomyces cerevisiae* [19], and bacteria [12, 20, 21]. The sequence of FGH contains a highly conserved cysteine site in different species, which suggests that FGH contains a serine esterase catalytic motif [12, 18, 22]. Human FGH can be detected in kidneys, liver, red blood cells, and most organs [23]. Its expression is often used as a genetic marker for diagnosis of retinoblastoma [24, 25], and it may also regulate cellular oxidative stress [26].

In this study, we investigated whether Skl interacts with FHG and further assessed whether FGH mediates the anti-oxidant activity of Skl.

## RESULTS

### Identification of proteins associated with short-form Klotho (Skl)

**Figure 1:**
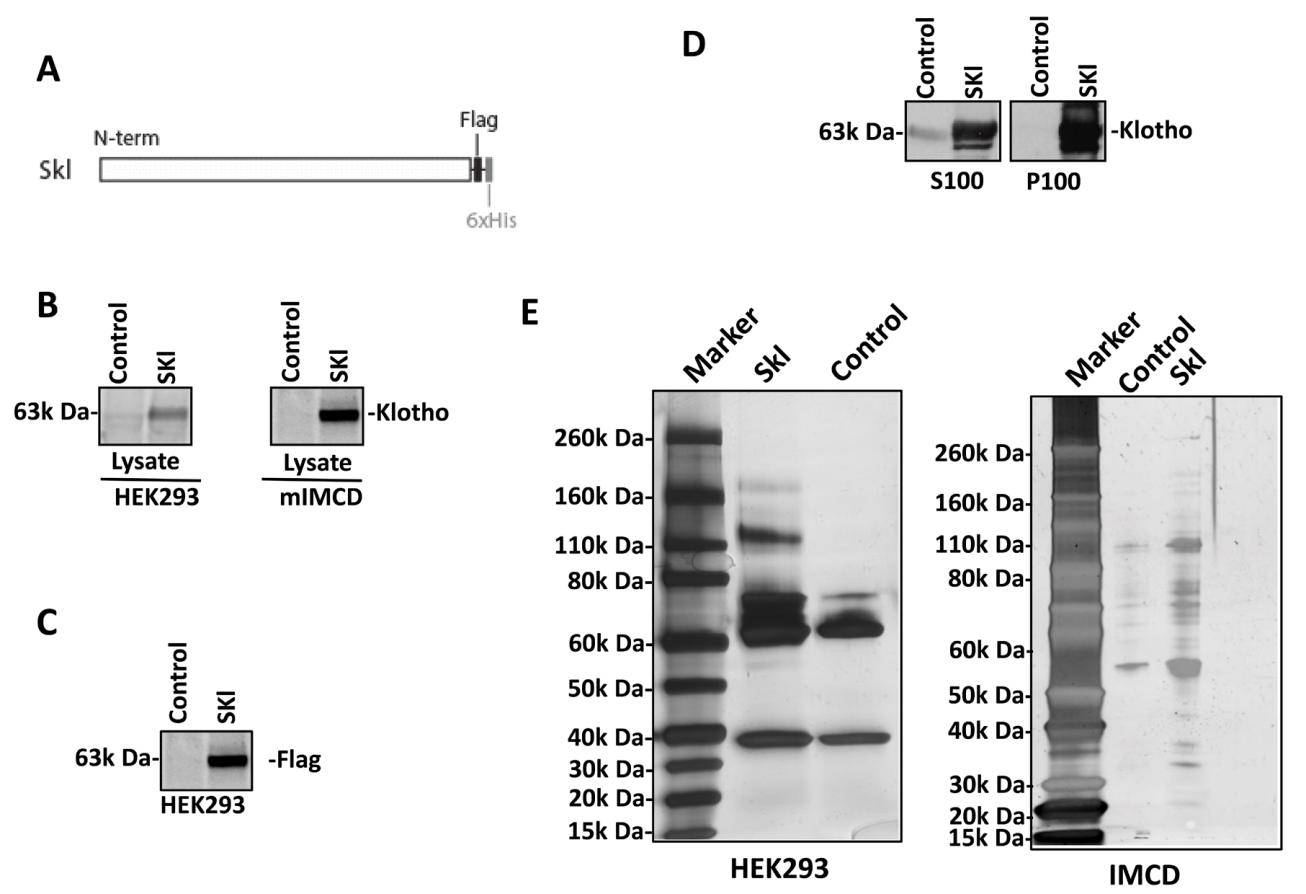
Secreted Klotho construction and expression **A.**, Schema for expression of recombinant Skl. Mouse Skl cDNA was fused with cDNA encoding C-terminal Flag and 6xHis and cloned into pAAV. **B.**, Presence of Skl. Recombinant Skl was expressed in HEK293 and mIMCD cells, and its expression was evaluated by western blotting using a Klotho antibody. **C.**, Flag tag expression. The recombinant Skl expressed in HEK293 cells was probed using Flag antibody to confirm the exogenous expression of Skl. **D.**, Subcellular localization of Skl . The HEK293 cell lysates with overexpression of Skl were separated by ultracentrifugation at 100,000 x g. The distribution of Skl in the membrane fraction (P100) and the cytosolic fraction (S100) were analyzed by western blotting using Klotho antibody. **E.**, Skl immune precipitation. Skl and associated proteins were co-immunoprecipated (co-IP) with Flag or Klotho antibody in 1% NP-40 lysis buffer. Co-IP products were subjected to stain-free gels from NEB.

The coding sequence of mouse short-form Klotho (Skl) was joined with the coding sequences for the Flag and 6xHis tags at the 3’ end, cloned into a pAAV vector, and driven by a CMV promoter to generate a recombinant Skl protein (Figure 1A). To determine the localization of Skl, we expressed recombinant Skl in HEK293 and mouse IMCD cells. After transfection for 48 h, the cell lysates were collected for western blot analysis of the Skl distribution. Skl was detected in the cell lysates of both HEK293 and IMCD cells (Figure 1B), indicating effective expression of Skl. Flag antibody was applied to confirm the distribution of the transgene-expressed Skl protein. As shown in Figure 1C, Flag-tagged Skl was found in HEK293 cells. To further confirm the subcellular localization of Skl, the membrane fraction (P100) and cytosolic fraction (S100) were separated by ultra-centrifugation in sucrose buffer and processed for western blot analysis of Skl. As shown in Figure 1D, recombinant Skl was distributed to both cytosolic and membrane fractions in HEK293 cells.

We further applied IP-coupled mass spectrometry to identify proteins associated with Skl, regardless of the sub-cellular localization. The cell lysates from HEK293 and mIMCD cells were incubated with Klotho or Flag antibody attached to the IP-Direct beads. The pull-down products were eluted with low-pH buffer from IP-Direct beads and subjected to SDS-PAGE to analyze the co-IP products using silver staining (Figure 1E). There were visibly different bands in the IP products between Skl-expressing cells and control cells. To identify these proteins, IP products were separated by SDS-PAGE and excised into different fragments, which were then digested with trypsin. The resulting peptides were analyzed by nano-LC-nano-ESI MS/MS and identified using Mascot software in the Swiss-Prot database. There were 46 proteins identified in Skl-overexpressing HEK293 cells compared with the control group. However, only 6 of 46 proteins were confirmed by the Flag antibody pull-down assay (Table 1). There were 16 proteins identified in Skl-overexpressing mIMCD cell lysates, but only 3 were confirmed by the Flag antibody pull-down assay (Table 2).

**Table 1:** Proteins associated with short-form klotho in HEK 293 cells

Swiss-Prot accession	Protein identity	Score	Matches	Number of unique peptides	Molecular Mass	Identify by Flag
ACTG_HUMAN	Actin, cytoplasmic 2 OS=Homo sapiens GN=ACTG1 PE=1 SV=1	1687	34	27	42108	
ACTN4_HUMAN	Alpha-actinin-4 OS=Homo sapiens GN=ACTN4 PE=1 SV=2	807	23	17	105245	+
MYH10_HUMAN	Myosin-10 OS=Homo sapiens GN=MYH10 PE=1 SV=3	714	27	18	229827	
POTEE_HUMAN	POTE ankyrin domain family member E OS=Homo sapiens GN=POTEE PE=1 SV=3	548	12	9	122882	
ACTN1_HUMAN	Alpha-actinin-1 OS=Homo sapiens GN=ACTN1 PE=1 SV=2	531	13	12	103563	
ACTC_HUMAN	Actin, alpha cardiac muscle 1 OS=Homo sapiens GN=ACTC1 PE=1 SV=1	509	16	13	42334	
KLOT_HUMAN	Klotho OS=Homo sapiens GN=KL PE=1 SV=2	338	8	8	116791	+
ESTD_HUMAN	S-formylglutathione hydrolase OS=Homo sapiens GN=ESD PE=1 SV=2	324	6	5	31956	+
MYH9_HUMAN	Myosin-9 OS=Homo sapiens GN=MYH9 PE=1 SV=4	156	8	6	227646	
RL6_HUMAN	60S ribosomal protein L6 OS=Homo sapiens GN=RPL6 PE=1 SV=3	122	2	2	32765	+
PHB2_HUMAN	Prohibitin-2 OS=Homo sapiens GN=PHB2 PE=1 SV=2	120	4	4	33276	+
SPTB2_HUMAN	Spectrin beta chain, non-erythrocytic 1 OS=Homo sapiens GN=SPTBN1 PE=1 SV=2	120	11	6	275237	
EF1G_HUMAN	Elongation factor 1-gamma OS=Homo sapiens GN=EEF1G PE=1 SV=3	101	2	2	50429	
EF2_HUMAN	Elongation factor 2 OS=Homo sapiens GN=EEF2 PE=1 SV=4	97	2	2	96246	
RTCB_HUMAN	tRNA-splicing ligase RtcB homolog OS=Homo sapiens GN=C22orf28 PE=1 SV=1	88	2	1	55688	
NONO_HUMAN	Non-POU domain-containing octamer-binding protein OS=Homo sapiens GN=NONO PE=1 SV=4	78	2	2	54311	+
H2B1B_HUMAN	Histone H2B type 1-B OS=Homo sapiens GN=HIST1H2BB PE=1 SV=2	72	2	1	13942	
SPTN1_HUMAN	Spectrin alpha chain, non-erythrocytic 1 OS=Homo sapiens GN=SPTAN1 PE=1 SV=3	68	9	3	285163	
RFA1_HUMAN	Replication protein A 70 kDa DNA-binding subunit OS=Homo sapiens GN=RPA1 PE=1 SV=2	67	4	2	68723	
RL5_HUMAN	60S ribosomal protein L5 OS=Homo sapiens GN=RPL5 PE=1 SV=3	64	2	2	34569	
PLST_HUMAN	Plastin-3 OS=Homo sapiens GN=PLS3 PE=1 SV=4	62	2	1	71279	
EFHD1_HUMAN	EF-hand domain-containing protein D1 OS=Homo sapiens GN=EFHD1 PE=1 SV=1	61	1	1	27025	
HNRDL_HUMAN	Heterogeneous nuclear ribonucleoprotein D-like OS=Homo sapiens GN=HNRPDL PE=1 SV=3	60	1	1	46580	
COR1C_HUMAN	Coronin-1C OS=Homo sapiens GN=CORO1C PE=1 SV=1	59	3	2	53899	
PABP1_HUMAN	Polyadenylate-binding protein 1 OS=Homo sapiens GN=PABPC1 PE=1 SV=2	59	2	1	70854	
SFPQ_HUMAN	Splicing factor, proline- and glutamine-rich OS=Homo sapiens GN=SFPQ PE=1 SV=2	57	1	1	76216	
IF2B1_HUMAN	Insulin-like growth factor 2 mRNA-binding protein 1 OS=Homo sapiens GN=IGF2BP1 PE=1 SV=2	56	2	1	63783	
HNRPM_HUMAN	Heterogeneous nuclear ribonucleoprotein M OS=Homo sapiens GN=HNRNPM PE=1 SV=3	45	2	1	77749	
EIF3G_HUMAN	Eukaryotic translation initiation factor 3 subunit G OS=Homo sapiens GN=EIF3G PE=1 SV=2	43	1	1	35874	
CH60_HUMAN	60 kDa heat shock protein, mitochondrial OS=Homo sapiens GN=HSPD1 PE=1 SV=2	42	2	1	61187	
CN166_HUMAN	UPF0568 protein C14orf166 OS=Homo sapiens GN=C14orf166 PE=1 SV=1	41	1	1	28165	
DNJA2_HUMAN	DnaJ homolog subfamily A member 2 OS=Homo sapiens GN=DNAJA2 PE=1 SV=1	38	1	1	46344	
PRDX6_HUMAN	Peroxiredoxin-6 OS=Homo sapiens GN=PRDX6 PE=1 SV=3	34	2	1	25133	
SERPH_HUMAN	Serpin H1 OS=Homo sapiens GN=SERPINH1 PE=1 SV=2	33	1	1	46525	
SERA_HUMAN	D-3-phosphoglycerate dehydrogenase OS=Homo sapiens GN=PHGDH PE=1 SV=4	32	1	1	57356	
XRCC5_HUMAN	X-ray repair cross-complementing protein 5 OS=Homo sapiens GN=XRCC5 PE=1 SV=3	31	1	1	83222	
RL32_HUMAN	60S ribosomal protein L32 OS=Homo sapiens GN=RPL32 PE=1 SV=2	27	2	1	15964	
ANXA5_HUMAN	Annexin A5 OS=Homo sapiens GN=ANXA5 PE=1 SV=2	27	1	1	35971	
GRP78_HUMAN	78 kDa glucose-regulated protein OS=Homo sapiens GN=HSPA5 PE=1 SV=2	25	2	2	72402	
ROA1_HUMAN	Heterogeneous nuclear ribonucleoprotein A1 OS=Homo sapiens GN=HNRNPA1 PE=1 SV=5	24	1	1	38837	
RTL1_HUMAN	Retrotransposon-like protein 1 OS=Homo sapiens GN=RTL1 PE=2 SV=3	22	1	1	155919	
IF2G_HUMAN	Eukaryotic translation initiation factor 2 subunit 3 OS=Homo sapiens GN=EIF2S3 PE=1 SV=3	20	2	2	51647	
PRS6B_HUMAN	26S protease regulatory subunit 6B OS=Homo sapiens GN=PSMC4 PE=1 SV=2	18	1	1	47451	
DDX5_HUMAN	Probable ATP-dependent RNA helicase DDX5 OS=Homo sapiens GN=DDX5 PE=1 SV=1	17	2	1	69618	
IQGA1_HUMAN	Ras GTPase-activating-like protein IQGAP1 OS=Homo sapiens GN=IQGAP1 PE=1 SV=1	17	1	1	189761	
MY18A_HUMAN	Unconventional myosin-XVIIIa OS=Homo sapiens GN=MYO18A PE=1 SV=3	15	1	1	234168	

**Table 2:** Secreted klotho associated proteins in mIMCD cells

Swiss-Prot accession	Protein identity	Score	Matches	Number of unique peptides	Molecular Mass	Identify in Flag
G3P_MOUSE	Glyceraldehyde-3-phosphate dehydrogenase OS=Mus musculus GN=Gapdh PE=1 SV=2	133	6	4	35787	
TBB5_MOUSE	Tubulin beta-5 chain OS=Mus musculus GN=Tubb5 PE=1 SV=1	100	6	4	49639	
HSP7C_MOUSE	Heat shock cognate 71 kDa protein OS=Mus musculus GN=Hspa8 PE=1 SV=1	76	7	4	70827	+
TBA1B_MOUSE	Tubulin alpha-1B chain OS=Mus musculus GN=Tuba1b PE=1 SV=2	61	6	4	50120	
CRYAB_MOUSE	Alpha-crystallin B chain OS=Mus musculus GN=Cryab PE=1 SV=2	56	1	1	20056	+
RS3_MOUSE	40S ribosomal protein S3 OS=Mus musculus GN=Rps3 PE=1 SV=1	51	4	3	26657	
ATPB_MOUSE	ATP synthase subunit beta, mitochondrial OS=Mus musculus GN=Atp5b PE=1 SV=2	50	1	1	56265	
RS18_MOUSE	40S ribosomal protein S18 OS=Mus musculus GN=Rps18 PE=1 SV=3	41	1	1	17708	
ESTD_MOUSE	S-formylglutathione hydrolase OS=Mus musculus GN=Esd PE=2 SV=1	38	2	2	31299	
RS10_MOUSE	40S ribosomal protein S10 OS=Mus musculus GN=Rps10 PE=1 SV=1	27	1	1	18904	+
RS7_MOUSE	40S ribosomal protein S7 OS=Mus musculus GN=Rps7 PE=2 SV=1	27	1	1	22113	
RS16_MOUSE	40S ribosomal protein S16 OS=Mus musculus GN=Rps16 PE=2 SV=4	27	1	1	16435	
GRIA1_MOUSE	Glutamate receptor 1 OS=Mus musculus GN=Gria1 PE=1 SV=1	24	1	1	101504	
EF1A1_MOUSE	Elongation factor 1-alpha 1 OS=Mus musculus GN=Eef1a1 PE=1 SV=3	17	2	1	50082	
ATPA_MOUSE	ATP synthase subunit alpha, mitochondrial OS=Mus musculus GN=Atp5a1 PE=1 SV=1	17	1	1	59716	
GPM6A_MOUSE	Neuronal membrane glycoprotein M6-a OS=Mus musculus GN=Gpm6a PE=1 SV=1	16	1	1	31128	

Among the candidate proteins that may be associated with Skl, *S*-formylglutathione hydrolase (FGH) received high scores and is present in both HEK293 and mIMCD cells. Therefore, FGH was chosen for further analysis. Several abundantly expressed proteins, most of which are of cytoskeletal or ribosomal origin, were detected and classified as nonspecific proteins.

### Co-localization of Skl and FGH

**Figure 2:**
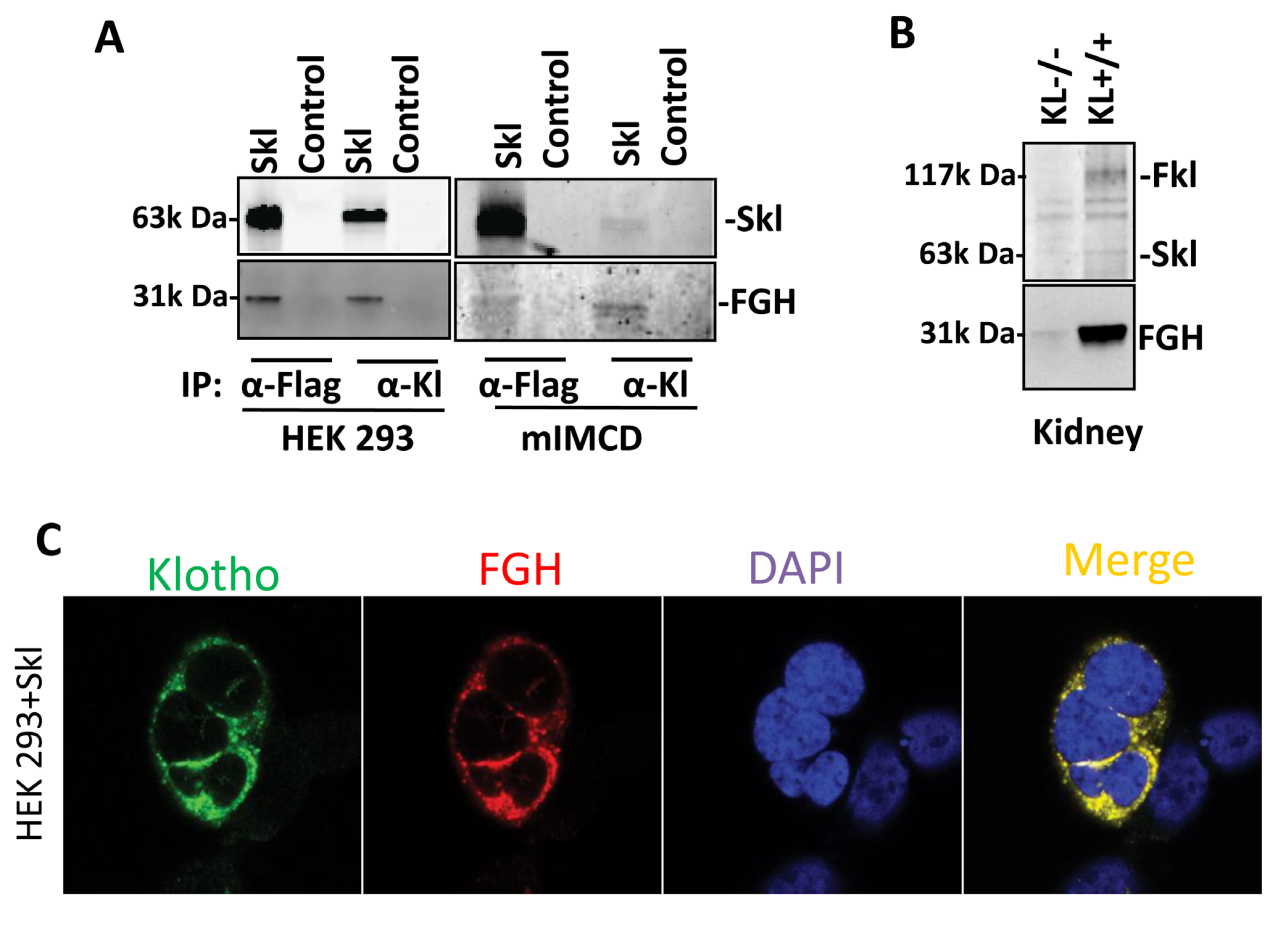
Identification of sKL-associated proteins **A.**, FGH was co-IPed with Skl. Recombinant Skl was pulled down from the lysates of HEK293 and mIMCD cells using Klotho or Flag antibody-conjugated beads (Thermo Fisher, Directly IP kit) at 4°C overnight. IP products were subjected to western blotting for detecting Skl and FGH using Klotho or FGH (ESD) antibodies, respectively. **B.**, Western blot analysis of Klotho IP products from *KL*^-/-^ and *KL*^+/+^ kidney lysates. Fkl and Skl (upper) and FGH (lower) were detected in *KL*^+/+^ IP products. However, Klotho or FGH was barely detectable in *KL*^-/-^ IP products. **C.**, Co-localization of FGH and Skl. HEK293 cells with overexpression of Skl were fixed on a coverslip. Klotho was labeled with Alexa 488-conjugated Flag antibody (Green); FGH was labeled with Alexa 555-conjugated anti-rabbit FGH antibody (Red); nuclei were labeled with DAPI (Blue).

To assess the association between FGH and Skl, we detected Klotho and FGH proteins in co-IP products from HEK293 or mIMCD cell lysates using Klotho or Flag antibodies. Western blot analysis indicated that Skl protein was always accompanied by FGH protein in the pull-down product in HEK293 and mIMCD cells, using either Flag or Klotho antibodies (Figure 2A). By contrast, the control cells did not show Klotho or FGH bands in the co-IP product from HEK293 or mIMCD cells (Figure 2A). These results support the mass spectrum result that Skl appears to be physically associated with FGH.

To assess the association between Skl and FGH proteins *in vivo*, we used wild-type (WT) and Klotho gene mutant (*KL*^-/-^) mice. [1] The Klotho gene is primarily expressed in kidney epithelial cells [27]. We performed a pull-down assay using Klotho antibody-conjugated IP-Direct beads in the kidney lysates from WT and *KL*^*-/-*^ mice. IP products were further subjected to western blotting analysis of Klotho and FGH. As shown in Figure 2B, neither Skl nor FGH were detected in the kidney IP products from *KL*^*-/-*^ mice. By contrast, both Skl and FGH were detected in the co-IP products from WT mice, suggesting that Skl is physically associated with FGH *in vivo*.

To further confirm this co-localization, we used immunofluorescence staining to identify the sub-cellular localization of Skl and FGH by confocal microscopy. As shown in Figure 2C, recombinant Skl was detected using the Flag antibody conjugated with Alexa 488 (Green), while endogenous FGH was detected using the FGH antibody conjugated with Alexa 555 (Red). The staining demonstrated a typical cytoplasm and membrane distribution for Skl (Figure 2C), which confirms cytosolic and membrane localization of Skl (Figure 1D). The merged photo shows that there is colocalization of Skl and FGH (yellow) around the nucleus (Figure 2C).

Therefore, the *in vitro* and *in vivo* results demonstrate that Skl may be physically associated with FGH and also that Skl is essential to the expression of FGH.

### Skl regulates FGH expression

**Figure 3:**
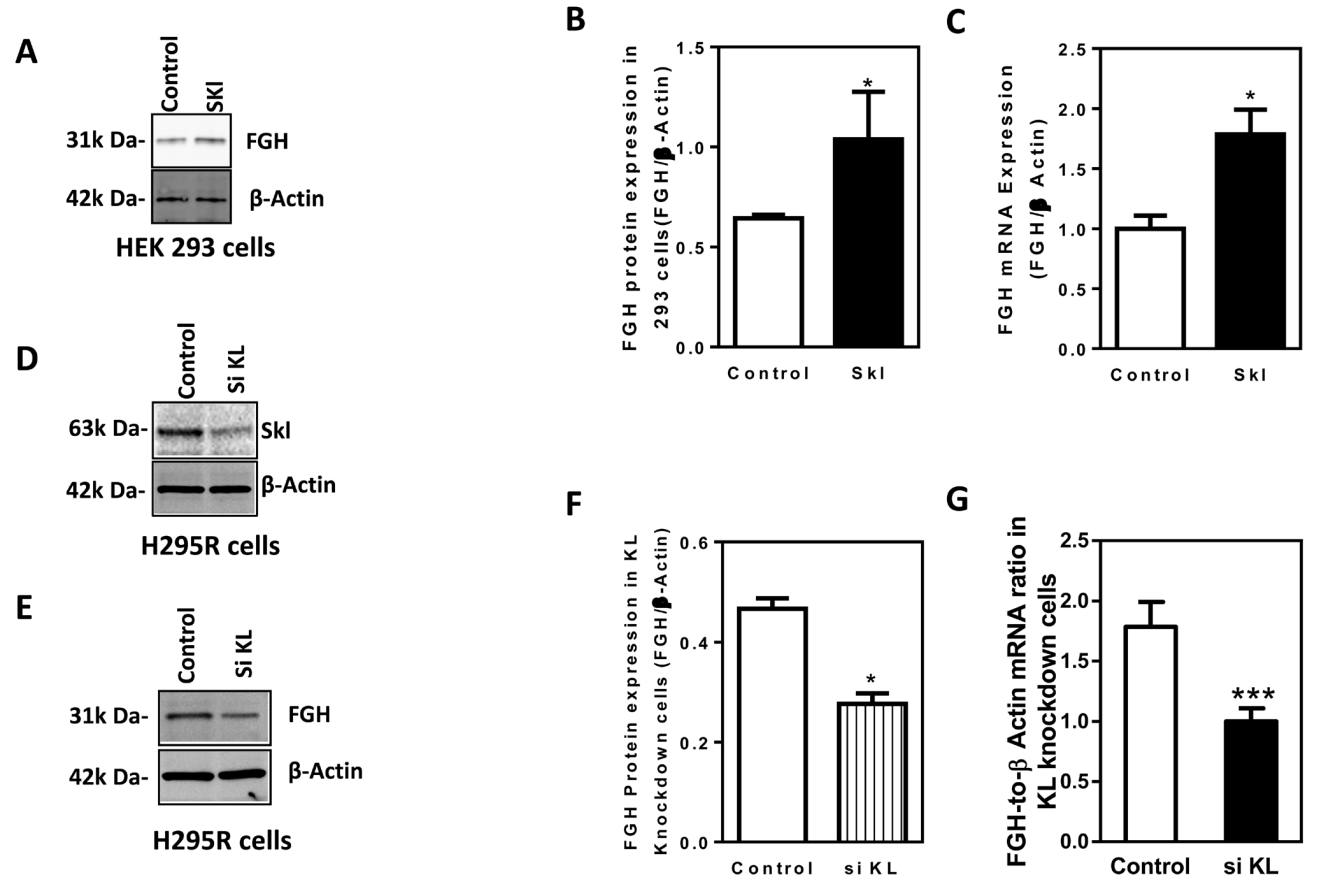
Skl regulates FGH expression **A.**, Western blotting analysis of FGH protein expression in HEK293 cells following overexpression of Skl. FGH protein expression was normalized to β-actin. **B.**, Quantification of FGH protein expression in HEK293 cells following overexpression of Skl. (*p < 0.05 *vs.* control, *N* = 3 independent experiments). **C.**, Real-time RT-PCR analysis of FGH mRNA expression in HEK293 cells following overexpression of Skl. (*p < 0.05 *vs.* control, *N* = 3). **D.**, Western blot analysis of Skl protein expression in H295R cells following silencing of Skl. **E.**, Western blot analysis of FGH protein expression in H295R cells following silencing of Skl. **F.**, Quantification of FGH protein expression in H295R cells following silencing of Skl. (*p < 0.05 *vs.* control). **G.**, Real-time RT-PCR analysis of FGH mRNA expression following silencing of Skl. (***p < 0.001 *vs.* control, *N* = 3). Bars, means ± SEM.

To determine whether Skl regulates FGH expression, we assessed the effects of overexpression and silencing of Skl on protein and mRNA expression of FGH in HEK293 cells. As shown in Figure 3A, 3B, overexpression of Skl increased FGH protein expression levels. Real-time QRT-PCR analysis indicated that FGH mRNA expression was also significantly increased by overexpression of Skl (Figure 3C), suggesting that Skl increases FGH expression, likely at the transcriptional level.

Since Skl expression is low in HEK293 cells, we used human adrenal cortical cells (NCI-H295R), which express robust levels of human Klotho and FGH simultaneously, to explore the effect of silencing of Skl on FGH expression. After 48 hours of transfection, Skl siRNA decreased Skl protein expression by 60% in H295R cells (Figure 3D), indicating effective silencing of Skl. FGH protein expression (Figure 3E, 3F) and mRNA expression (Figure 3G) were reduced significantly by silencing of Skl. Therefore, Skl appears to regulate FGH expression by regulating FGH gene expression.

### Kid3 mediates the effect of Skl on FGH expression

**Figure 4:**
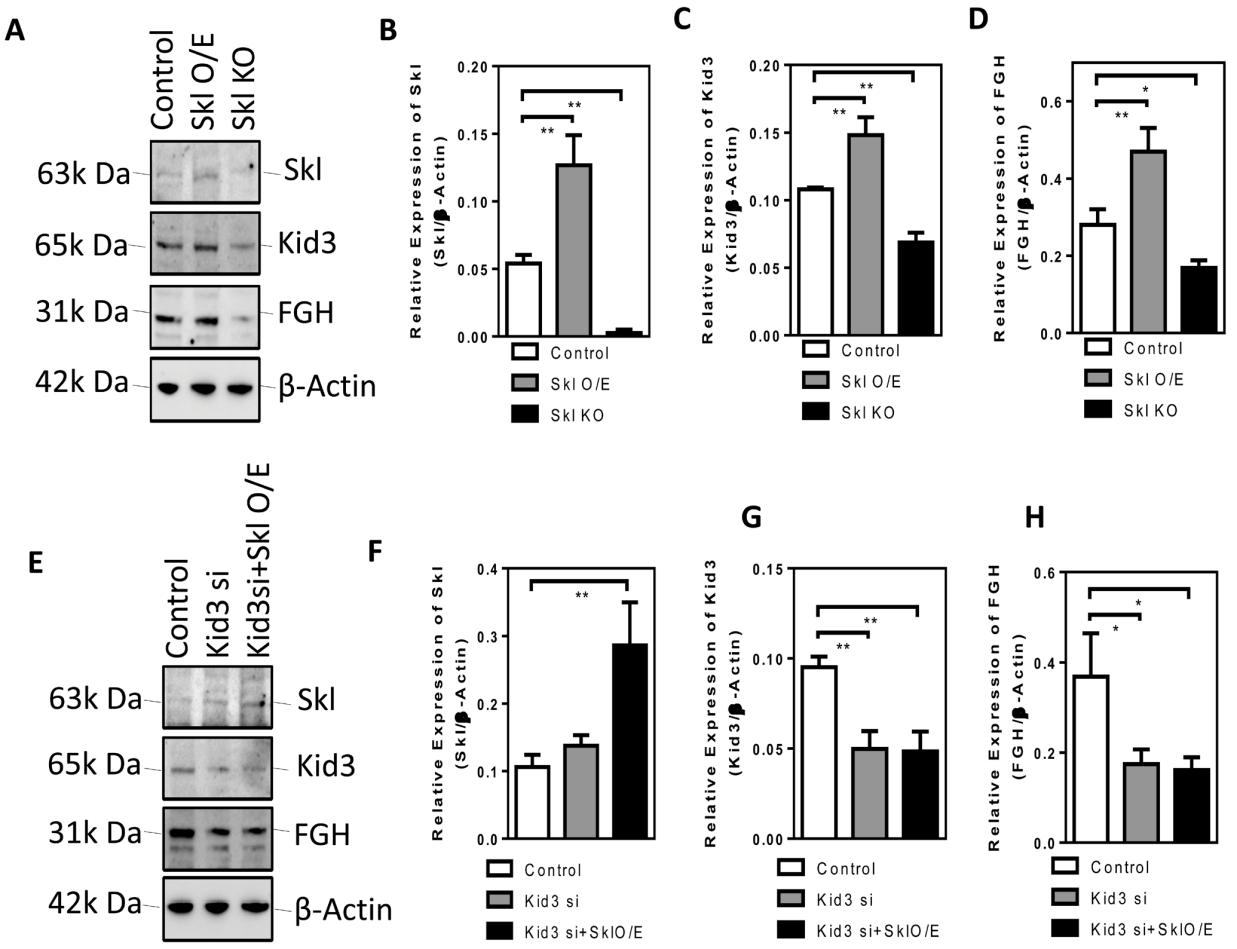
Kid3 mediates the effect of Skl on FGH expression **A.**, Western blot analysis of Skl, Kid3 and FGH in DCT cells following overexpression of Skl. Quantification of Skl **B.**, Kid3 **C.**, and FGH **D. E.**, Western blot analysis of Skl, Kd3 and FGH in DCT cells following silencing of Kid3 and overexpression of Skl. Quantification of Skl, Kid3 and FGH protein expression. Bars, means ± SEM.

To further assess how Skl regulates FGH expression, we looked into several transcription factors (e.g., Kid3) that may be involved in FGH expression. We found that overexpression of Skl increased Kid3 expression while knockout of Skl using the Crispr/Cas9 system suppressed Kid3 expression (Figure 4A-4D). These results suggest that Skl regulates Kid3 expression.

Next, we assessed whether Kid3 mediates the effect of Skl on FGH expression. Silencing of Kid3 decreased FGH expression and abolished the promoting effect of overexpression of Skl on FGH expression (Figure 4E-4H). These results suggest that Kid3 is an important mediator of Skl-induced FGH expression.

### Klotho gene mutation suppresses FGH expression and causes anemia and kidney damage in mice

**Figure 5:**
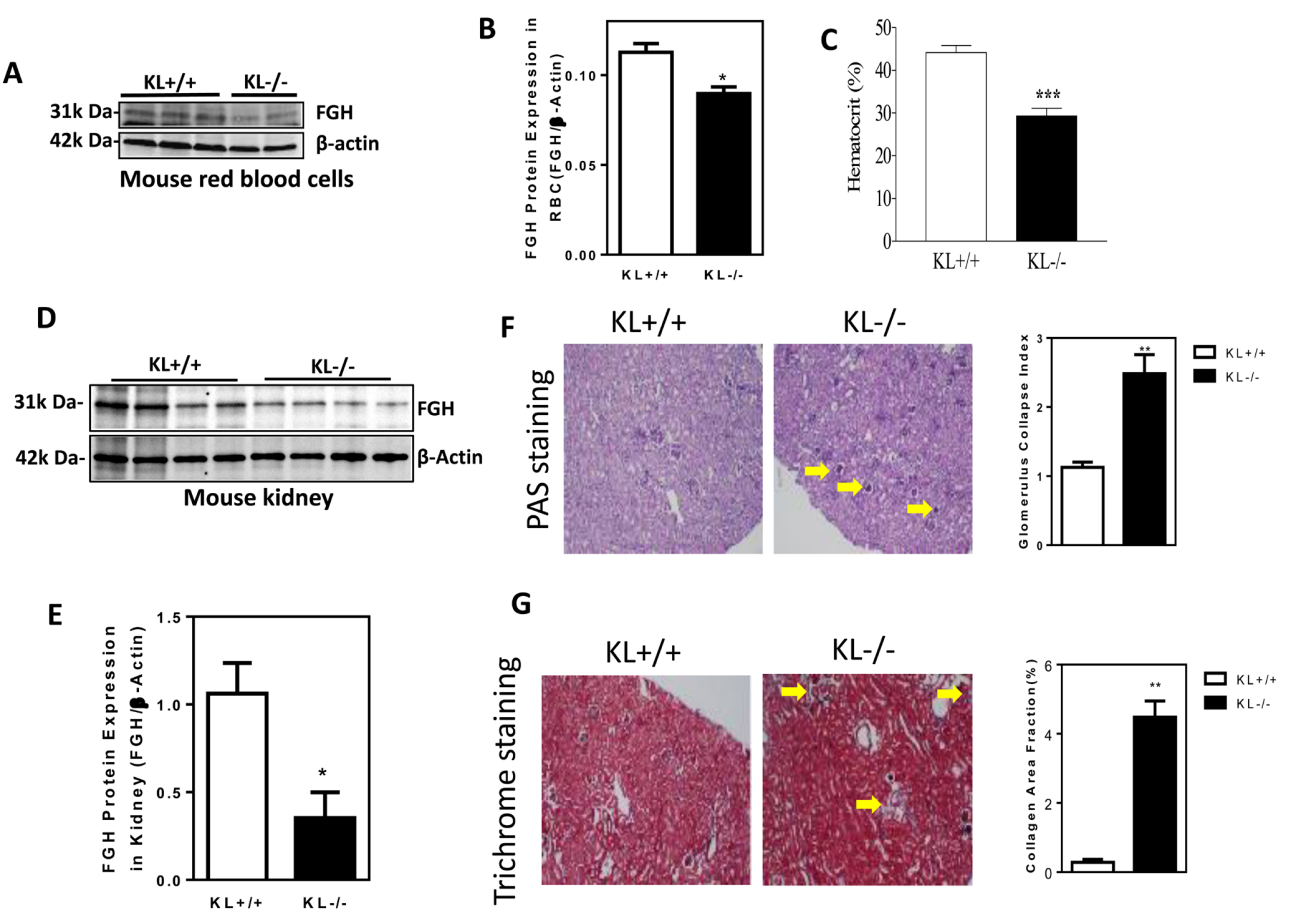
Klotho gene mutation suppresses FGH expression and causes anemia and kidney damage in mice **A.**, Western blot analysis of FGH expression in red blood cells of *KL*^*-/-*^ *mice.* **B.**, Quantification of FGH expression in red blood cells (*p < 0.05, *vs. KL*^*+/+*^ mice, *N* = 6). **C.**, Hematocrit (***p < 0.001 *vs. KL*^*+/+*^ mice, *N* = 5). **D.**, Western blot analysis of FGH expression in the kidney cortex of *KL*^*-/-*^ mice. **E.**, Quantification of FGH expression in the kidney cortex (*p < 0.05 *vs. KL*^*+/+*^ mice, *n* = 4). **F.**, PAS staining of glomerular matrix and semi-quantification of glomerular collapse (**p < 0.01 *vs. KL*^*+/+*^ mice, *N* = 4). **G.**, Trichrome staining of collagen deposition in kidneys and semi-quantification of collagen area fraction (**p < 0.01 *vs. KL*^*+/+*^ mice, *N* = 4). Bars, means ± SEM.

To assess the *in vivo* effect of Klotho gene deficiency, we collected kidneys and red blood cells from *KL*^*-/-*^ mice and age-matched WT mice. FGH protein expression was decreased significantly in red blood cells in *KL*^*-/-*^ mice relative to WT mice (Figure 5A, 5B), suggesting that Klotho deficiency attenuated FGH expression. Hematocrit was significantly decreased in *KL*^*-/-*^ mice (Figure 5C), indicating that Klotho deficiency caused anemia.

FGH protein expression was decreased significantly in kidneys in *KL*^*-/-*^ mice relative to WT mice (Figure 5D, 5E), suggesting that Klotho deficiency attenuated FGH expression. Notably, obvious glomerular collapse (Figure 5F, 5G) and collagen deposition (Figure 5H, 5I) were found in kidneys of *KL*^*-/-*^ mice, indicating that Klotho deficiency caused kidney damage.

Thus, Klotho appears to regulate FGH expression *in vivo* which is involved in the maintenance of normal homeostasis of red blood cells and kidneys.

### Skl exerts antioxidant activity *via* FGH

**Figure 6:**
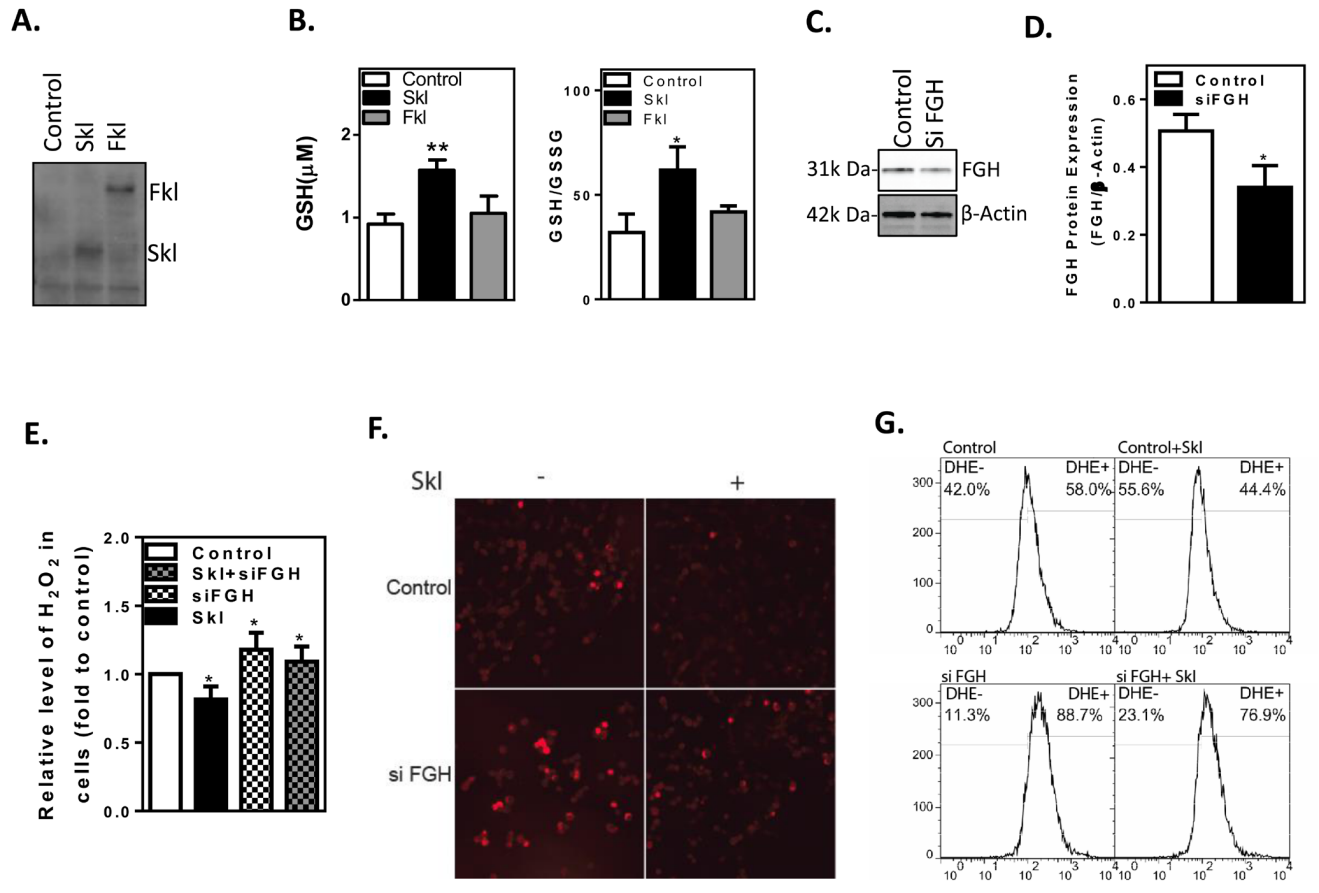
Skl regulates cellular anti-oxidant capacity **A.**, Western blot analysis of full-length Klotho (Fkl) and short-form Klotho (Skl) proteins in HEK293 cells. **B.**, Intracellular GSH levels and the GSH/GSSG ratio were increased by Skl. (*p < 0.05 *vs*. control). **C.**, Western blot analysis of FGH protein expression. **D.**, Quantitative analysis of FGH protein expression. (*p < 0.05 *vs*. control). **E.**, H_2_O_2_ released by the cells was measured by Amplex Red reagent. (*p < 0.05 *vs*. control). **F.**, DHE staining of the O_2_^-^ level. **G.**, Flow cytometry analysis of DHE-positive cells. Skl reduced the O_2_^-^ level (upper panel). Knockdown of FGH raised the O_2_^-^ level, regardless of Skl expression (lower panel). Bars, means ± SEM.

FGH is a key catalytic enzyme in the generation of glutathione (GSH) from S-formylglutathione and H_2_O. GSH and its associated enzymes are the major antioxidant system in the cell. The ratio of GSH to oxidized glutathione (GSSG) within the cell is a marker of cellular antioxidant capacity [28]. Full-length Klotho (Fkl) was reported to have a suppressor effect on oxidative stress [27]. Therefore, we evaluated the effect of overexpression of Fkl and Skl on the GSH/GSSG ratio in HEK293 cells. As shown in Figure 6A, Fkl and Skl were overexpressed in HEK293 cells. The ratio of GSH/GSSG was determined by a luminescence-based assay (GSH/GSSG-Glo Assay, Promega). Skl increased the GSH level and the ratio of GSH/GSSG by ∼50%, while Fkl did not affect the GSH level or the ratio of GSH/GSSG significantly (Figure 6B). Therefore, Skl was the subject of the remaining study.

To determine whether FGH mediates the antioxidant activity of Skl, we knocked down FGH in HEK293 cells by transfecting FGH siRNA duplexes. After 48 h of transfection, FGH protein expression was determined by western blot (Figure 4C). The siRNA transfection reduced FGH expression by 45% (Figure 6C, 6D). We further evaluated intracellular H_2_O_2_ levels using the Amplex Red assay following silencing of FGH. Overexpression of Skl decreased intracellular H_2_O_2_ levels, while silencing of FGH increased intracellular H_2_O_2_ levels (Figure 6E). Interestingly, silencing of FGH abolished the attenuating effect of Skl on intracellular H_2_O_2_ (Figure 6E). These results suggest that Skl decreases H_2_O_2_ levels by increasing FGH expression and the GSH/GSSG ratio.

The major source of H_2_O_2_ is superoxide (O_2_). A decrease in H_2_O_2_ facilitates conversion of O_2_^-^ to H_2_O_2_. Thus, DHE staining was used to assess the level of intracellular O_2_^-^ (Figure 6F). Flow cytometry was employed to quantify DHE-positive cells. The percentage of DHE-positive (+) and DHE-negative (-) cells was 58% D and 42%, respectively, in the control HEK293 cells (Figure 6G). Overexpression of Skl reduced DHE-positive cells to 44% (Figure 6F, 6G), suggesting that Skl decreases intracellular superoxide levels. On the other hand, silencing of FGH resulted in an increase in O_2_^-^ levels (Figure 6F, 6G). The flow cytometry analysis showed that knockdown of FGH increased DHE-positive cells from 58% to 88.7% (Figure 6G). Following silencing of FGH, overexpression of Skl failed to decrease the percentage of DHE-positive cells to the control level (Figure 6F, 6G), indicating that knockdown of FGH largely blocked the attenuating effect of Skl on intracellular O_2_^-^. These results suggest that the antioxidant activity of Skl is primarily mediated by FGH through the GSH/GSSG-H_2_O_2_-O_2_^-^ pathway.

### N-glycosylation of Skl

**Figure 7:**
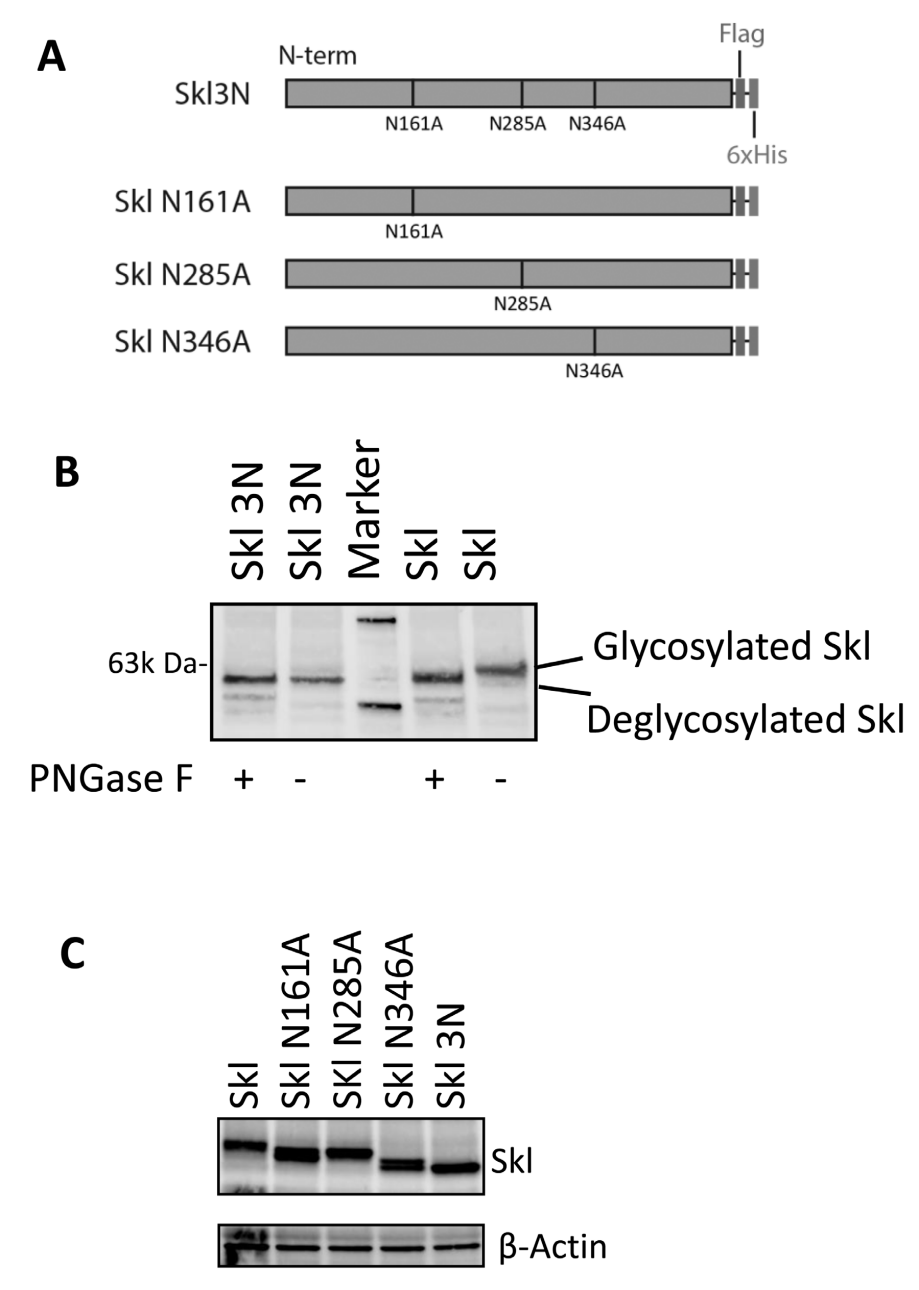
N-glycosylation of Skl **A.**, Schemes for recombinant Skl mutations. The potential glycosylation sites were mutated in combination or individually. **B.**, Western blot analysis of N-glycosylation of Skl. Native or three point mutations in Skl were expressed in HEK293 cells and treated or not treated with PNGase F, as indicated. The apparent *M*r was evaluated using Klotho antibody. **C.**, N-glycosylation site identification. Individually mutated Skl genes were expressed in HEK293 cells. Western blot analysis of cell extracts from mutated or native Skl. All the mutant Skl proteins show shifted *M*r.

Since there is co-localization of Skl and FGH, we further assessed the potential functional interaction between these two molecules. Based on analysis of the amino acid sequence of Skl, asparagines at sites N161A, N285A, and N346A are potentially *N*-glycosylation sites. To assess whether these sites are indeed *N*-glycosylated, we mutated these three sites to alanine (the single mutations are denoted as SklN161A, SklN285A, and SklN346A and the triple mutation as Skl3N; Figure 7A). To confirm N-glycosylation, Skl and Skl3N were expressed in HEK293 cells, and the cell lysates were treated with or without PNGase F, which specifically cleaves *N*-glycan side chains from proteins. The glycosylated and de-glycosylated Skl should show different apparent mobilities with SDS-PAGE [29-31]. Skl3N showed a consistent apparent relative molecular weight (*M*r), regardless of the presence or absence of PNGase F (Figure 7B). However, PNGase F-treated Skl showed different apparent *M*r than non-treated Skl (Figure 7B). In addition, PNGase F-treated Skl showed the same apparent *M*r as Skl3N (Figure 7B). These results suggest that Skl is *N*-glycosylated during post-translational modification.

To further confirm the glycosylation sites, individual mutants were expressed in cells. All mutants showed a different apparent *M*r from Skl and de-glycosylated Skl (Skl3N, Figure 7C). This result suggests that all three sites were glycosylated.

### N-glycosylation determines Skl activity

**Figure 8:**
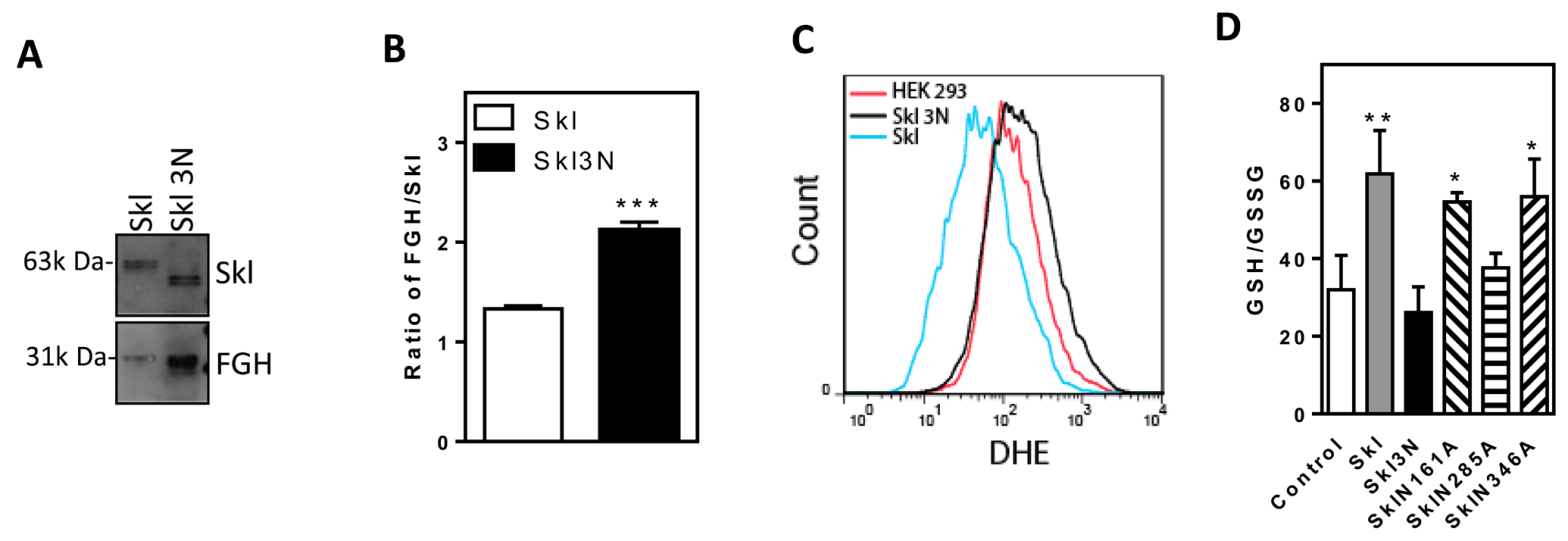
N-glycosylation regulates Skl activity **A.**, Western blot analysis of IP products from native Skl or Skl mutants with three point mutations. The products were probed with Klotho or FGH antibodies separately. **B.**, The ratios of FGH to Skl (***p < 0.01 *vs.* Skl). **C.**, Flow cytometry analysis of O_2_^-^ (DHE) levels. **D.**, The GSH/GSSG ratio following expression of Skl mutants (*p < 0.05, **p < 0.01 *vs.* control). Bars, means ± SEM.

To determine the function of Skl glycosylation, we evaluated the binding of FGH to SKl and de-glycosylated Skl3N. The binding of FGH to Skl3N was increased relative to the binding of FGH to Skl (Figure 8A). The FGH/Skl3N ratio was nearly doubled *vs.* that of FGH/Skl (Figure 8B). The increased binding of FGH to Skl3N is likely a compensatory response to Skl3N inactivity.

To further evaluate the function of glycosylation of Skl, we assessed the effect of overexpression of Skl and Skl3N on intracellular O_2_^-^ levels in HEK293 cells. Skl attenuated DHE staining intensity (Figure 8C), indicating a decrease in intracellular O_2_^-^ levels. Mutation of Skl (Skl3N), however, increased DHE staining intensity, indicating that Skl3N enhances the intracellular O_2_^-^ level (Figure 8C). The increased O_2_^-^ level is due to a decrease in the antioxidant capacity of the mutated Skl (Skl3N). These results suggest that N-glycosylation of Skl determines its interaction with FGH and its antioxidant activity.

### Glycosylation at site 285 confers Skl antioxidant activity

To further assess the specific and key site that determines the antioxidant activity of Skl, we evaluated the GSH/GSSG ratio following expression of individual mutants. As shown in Figure 8D, Skl increased the GSH/GSSG ratio. Mutation of Asp at site 285 led to a loss of Skl activity in the regulation of the GSH/GSSG ratio, which was similar to mutation of all three glycosylation sites (Skl3N). This result suggests that N-glycosylation of the Asp at residue 285 is likely the key site that determines the antioxidant activity of Skl. Therefore, FGH function may be regulated by Skl *via* N-glycosylation at residue 285.

## DISCUSSION

The major finding of this study is that Skl regulates FGH expression and activity. FGH is a key enzyme in the biosynthesis of GSH, which is the major endogenous anti-oxidant generated by the cells (S-formylglutathione + H_2_O → glutathione + formate). GSH participates directly in the neutralization of free radicals (e.g., H_2_O_2_, O_2_^-^) and reactive oxygen compounds as well as maintains exogenous antioxidants, such as vitamins C and E, in their reduced (active) forms [32-35]. FGH also appears to be part of a formaldehyde detoxification pathway that is universal [36, 37]. We found that overexpression of Skl increased anti-oxidant capacity, as evidenced by increases in total GSH and the GSH/GSSG ratio and decreases in intracellular H_2_O_2_ and O_2_^-^ (Figure 6). This beneficial effect appears to be mediated by FGH, because silencing of FGH abolished the effect of overexpression of Skl on GSH and intracellular H_2_O_2_ and O_2_^-^ (Figure 6).

Skl may positively regulate FGH expression (Figure 3). The regulation of FGH by Skl may occur at the transcriptional (mRNA) level. We further found that Skl regulated FGH likely *via* Kd3, a transcription factor. The finding that Skl regulates FGH expression is interesting and significant, as it provides new insight into the regulation of anti-oxidant capacity, which is critical for cell survival. Klotho deficiency decreased FGH expression and activity, leading to a decrease in anti-oxidant capacity. This finding was validated in red blood cells (RBCs) in Klotho gene mutant (*KL*^-/-^) mice (Figure 5). Kidneys are the major source of Klotho in the circulation [27]. Klotho gene deficiency in kidneys leads to a decrease in plasma Skl which suppresses FGH expression in RBCs. GSH is the primary anti-oxidant that protects red blood cells. A decrease in the GSH levels or the GSH/GSSG ratio would damage red blood cells, leading to anemia. Indeed, *KL*^-/-^ mice developed anemia as evidenced by a significant decrease in hematocrit (Figure 5). The suicidal death of erythrocytes may be partly due to Klotho deficiency-induced suppression of FGH expression in *KL*^*-/-*^ mice (Figure 5). This study showed that FGH is also expressed in kidneys and may be regulated by Klotho (Figs. 2&5). Klotho deficiency (*KL*^*-/-*^) diminished FGH expression (Figure 2&5). A decrease in FGH would decrease the antioxidant capacity which causes kidney damage. Indeed, *KL*^*-/-*^ mice demonstrated glomerular collapse and interstitial fibrosis (Figure 5). Haplodeficiency of Klotho gene (*K*^*+/-*^) also impairs kidney function [27, 38, 39]. The kidney damage may also contributes to Klotho deficiency-associated anemia. Chronic kidney disease (CKD) is associated with a significant decrease in renal Klotho expression [27]. Plasma levels of Klotho is also decreased in patients with CKD. In fact, a decrease in plasma Klotho has been recommended as a biomarker of CKD [27].

Another interesting finding is that Skl may directly interact with FGH and regulate its function in the generation of GSH. Using IP-coupled mass spectrometry and protein identification techniques, we found that Skl may be physically associated with FGH in HEK293 cells and IMCD cells (Tables 1 and 2, Figure 2). Importantly, this association was also validated in the kidneys (Figure 2). Further analysis confirmed that Skl and FGH were co-localized in the cytosol (Figure 2). *N*-glycosylation is the most common type of glycosylation in eukaryotic cells, which facilitates protein folding, protein-protein interactions, and cell attachment. Skl is *N*-glycosylated during the post-translational modification process [5], but the exact glycosylation sites have never been reported. We demonstrated that there are three *N*-glycosylation sites on Skl that were glycosylated simultaneously (Figure 7). Interestingly, Skl may functionally interact with FGH through N-glycosylation, because mutation of *N*-glycosylation sites on Skl abolished its enhancing effect on GSH levels and the GSH/GSSG ratio (Figure 8). This novel finding also suggests that *N*-glycosylation is a critical mechanism that mediates the regulation of FGH activity by Skl. We further demonstrated that N285 is the key site that determines the biological activity of Skl.

We previously reported that overexpression of full-length Klotho (Fkl) attenuates oxidative stress in aortic smooth cells [40]. The anti-oxidative effect of Fkl cannot be due to GSH/GSSG, which was not altered by overexpression of Fkl (Figure 6A, 6B). By contrast, Fkl downregulated Nox2 NADPH oxidase protein expression, contributing to its suppressor effect on superoxide generation [40].

The protein identification results showed that Skl binds to multiple proteins, most of which are chaperone proteins or ribosomal proteins (Tables I and II). In particular, Skl binds to heat shock proteins 60 and 70. As chaperone proteins, heat shock proteins bind to functional proteins and facilitate folding and post-translational modification. Therefore, we infer that Skl regulates post-translational modification and protein homeostasis, which warrants further investigation.

In summary, this study demonstrates for the first time that Skl physically and functionally interacts with FGH, which regulates cellular anti-oxidant activity. In addition, Skl also regulates the expression of FGH, a critical enzyme for the generation of GSH, a major antioxidant that is ubiquitously distributed in different cellular compartments in all cells. These results partially explain why Klotho gene mutation causes extensive premature aging phenotypes in nearly all organs or tissues [1, 27]. Overexpression of Klotho enhances FGH expression and GSH levels, which may contribute to extended lifespan [2, 27].

## MATERIALS AND METHODS

### Cell culture and transfection

Mouse IMCD cells, human HEK293 cells, and human adrenal cortical carcinoma NCI-H295R cells were maintained in Dulbecco’s modified Eagle’s F-12 medium (ATCC) or Dulbecco’s modified Eagle’s medium (ATCC), containing 10% fetal bovine serum. For transfections, cells were cultured in 96-, 12-, or 6-well plates or 10-cm dishes and transfected 24 h after plating at 80- 90% confluence using Lipofectamine 2000 (Invitrogen) according to the manufacturer’s instructions.

### Construction and expression of recombinant mouse Skl

For generating a Skl expression vector, Skl cDNA was amplified from the pAAV-mKL vector [40] using the oligonucleotides Skl5 and Skl3 (Supplementary Table S1). A sequence corresponding to the last 15 amino acids of Skl, which is different from Fkl, was inserted into the anti-sense primer Skl3, cloned into the pCR4-TOPO vector (Invitrogen), excised using KpnI and XbaI sites, and ligated into the similarly digested pAAV-mKL vector. The insert fragment was sequenced using vector-based primers and found to match that of the gene model. Flag and 6xHis coding sequences were annealed by the two oligonucleotides F6H5 and F6H3 (Supplementary Table S1). A stop codon was inserted into primer F6H3 to block transcription. The annealed product was inserted into pAAV-Skl between the XbaI and HindIII sites to generate pAAV-SklF6H. For establishing a stable cell line that expresses Skl, the coding sequence was excised using EcoRI and XhoI sites and ligated into the similarly digested pCDNA3 vector.

We also generated de-glycosylated Skl. Briefly, a dsDNA fragment was synthesized (Genscript), which corresponds to the coding sequence of Skl but mutated at three predicted asparagine sites (positions 161, 285, and 346) to alanine. The mutated fragments were excised for replacing the corresponding coding sequence on the pAAV vector to generate single and triple mutations.

### Immunoprecipitation

HEK293 and IMCD cells, which expressed recombinant Skl-Flag-6xHis, were rinsed with cold PBS twice, followed by protein extraction using cell extraction buffer (50 mM Tris-HCI, pH 7.4, 0.5% Triton X-100, and protease inhibitors). Protein concentration was measured using the BCA Protein Assay Reagent (Thermo Fisher). Recombinant Skl was pulled down with the Direct IP Kit (Thermo Fisher) using Klotho or Flag antibodies.

### In-gel protein identification

Coomassie Blue-stained bands were excised from the gel, cut into pieces, and fully destained with 50 mM Na_2_S_2_O_3_, 15 mM K3 [Fe(CN)_6_], and then 25 mM (NH_4_)HCO_3_ in 50% acetonitrile. Gel pieces were reduced in 55 mM TCEP (Thermo Fisher), 25 mM (NH_4_)HCO_3_ at 60°C for 10 minutes. Gel pieces were alkylated by adding 100 mM iodoacetamide (Thermo Fisher) and 25 mM (NH_4_)HCO_3_ and incubated at room temperature in the dark for 1 hour. After removing the alkylation buffer, the gel pieces were washed twice with 25 mM (NH_4_)HCO_3_ in 50% acetonitrile, then dehydrated with 100% acetonitrile. Trypsin (100 ng) in 10 µl of 25 mM (NH_4_)HCO_3_ was added before an additional 25 µl of 25 mM (NH_4_)HCO_3_ was added to cover the gel pieces. These were then incubated at 30°C overnight, and 25 µl of 100% acetonitrile was added to the digest solution containing peptides, which was then completely dried using a speed-vac.

### HPLC/MS/MS and MASCOT database search

An aliquot of the trypsin digest was analyzed with a Dionex UltiMate 3000 liquid chromatography system and an ABI MDS Sciex Qstar Elite LC/MS/MS system. The peptide mixture was loaded onto an Acclaim PepMap100 C18 column. The peptides were then separated with a discontinuous gradient of 0.01% TFA, 0.09% formic acid, 2-80% acetonitrile at a flow rate of 200 nl/min. MS/MS data was collected using ABI Analyst QS 2.0 software by fragmentation of ions between 300 and 2500 m/z and having a charge between +2 and +3. For each ion selected, fragmentation data was collected for a maximum of 5 seconds and, once selected, was excluded from reselection for 250 seconds. The data collected were submitted to our in-house MASCOT (Matrix Science) server.

### Western blotting

Western blotting was performed as described previously [41-44]. Briefly, soluble extracts were combined with 4×SDS sample buffer, boiled for 1 minute, and electrophoresed on a 4-15% gradient Criterion gel (Bio-Rad). Gels were transferred to nitrocellulose membranes using a Trans-Blot Turbo Transfer System (Bio-Rad), probed with primary and fluorescent secondary Abs, and scanned on a ChemiDoc MP Image system. The antibodies used were: anti-Flag M2 (Sigma-Aldrich), anti-Klotho (R&D Systems), anti-ESD (Abcam), and ESD Polyclonal Antibody (Pierce).

### Immunofluorescence microscopy

Immunofluorescence staining was performed as we described previously [38, 41, 42]. Briefly, cells were seeded with glass coverslips into 6-well plates and transfected with a constructed pAAV-SklF6H vector for 2 days. At the end of culturing, cells were washed in cold PBS and fixed in cold 4% paraformaldehyde for 15 minutes at room temperature. Fixed cells were washed using PBS and permeabilized by incubation with 0.25% Triton X-100 for 5 minutes. The cells were then washed in PBS, blocked using 10% BSA in PBS, and incubated with anti-FLAG M2 antibody (1:500) and ESD antibody (1:500) in incubation buffer (3% BSA in PBS) overnight at 4°C. Cells were washed in PBS and incubated with Alexa 568-conjugated (1:1,000) or Alexa 488-conjugated (1:1,000) secondary antibodies (Invitrogen) in incubation buffer for 1 hour at room temperature. The cells were then washed in PBS and mounted with Ultra Cruz Mounting Medium containing DAPI (Santa Cruz Biotechnology) and cured for at least 24 hours at room temperature in the dark. Images were taken using a Nikon Eclipse Ti confocal microscope.

### Analysis of Kid3 in the regulation of FGH by Skl

To knockout Skl, we employed Crispr/Cas9 system from Santa Cruz to establish the stable cell line [45, 46]. CRISPR/Cas9 KO Plasmid (sc-421290) and Klotho HDR Plasmid (sc-421290-HDR) were co-transfected into DCT cells for 24h. Puromycin was loaded into cell medium for screening positive cells. Following selection, single cell colonies were seeded into 96-well plates for culture. The colony was used for confirming knockout of Skl and was cultured as a stable cell line. To overexpress Skl, we transfected pcDNA-Skl-Flag (1 µg) into DCT cells cultured in 6-well plates for 48h. The whole cell lysates was used for assessing Skl, Kid3 and FGH expression by western blot. DCT cells transfected with pcDNA3 plasmid served as a control.

Kid3 expression was suppressed by a siRNA complex from SantaCruz (ZNF354C siRNA). Kid3 siRNA complex (2 µM) and Skl overexpression plasmids (1 µg) were co-transfected into DCT cells in 6-well plate for 48h. DCT cells transfected with Control siRNA-A and pcDNA3 plasmids served as s control. The cell lysate was collected for western blot analysis of Skl, FGH and Kid3 expression.

### Measurement of O_2_- and H_2_O_2_

The intracellular O_2_^-^ level was assessed by dihydroethidium (DHE) fluorescence analysis, as described previously [47-49]. Briefly, the attached cells were resuspended in DMEM containing 10% FBS, washed twice with PBS, and incubated with DHE at 37°C, in the dark, for 30 min. After removal of staining buffer, the cells were washed with PBS. Flow cytometry analysis was carried out on a FACS Calibur instrument. H_2_O_2_ was measured using the Amplex Red method*.* Briefly, the cells were washed once with phosphate-buffered saline (PBS) and subsequently incubated in PBS containing Amplex Red (Invitrogen). H_2_O_2_ was measured with the Synergy 2 plate reader (Biotek), with absorbance excitation/emission at 530 nm/590 nm, according to the manufacturer’s instructions. The readings were then normalized by the number of cells for each condition.

### GSH/GSSG ratio assay

Cells were cultured in 96-well plates for 12 h and transfected with Skl or mutated Skl for 2 days. The ratio of reduced glutathione (GSH) and oxidized glutathione (GSSG) were analyzed by using the GSH/GSSG Glo Assay (Promega, Madison, USA) following the manufacturer’s instructions. The luminescence signal was measured with a Synergy 2 plate reader (Biotek). The GSH/GSSG ratio was calculated in accordance with the procedure provided.

### QRT-RT-PCR

The QRT-RT-PCR procedure was performed as described previously [41]. Briefly, total RNA was extracted from cultured cells using TRIzol reagent (Invitrogen) and the Turbo DNA-free kit (Invitrogen). RNA quality and quantity were measured using a NanoDrop spectrophotometer. Total RNA was transcribed to DNA using a Superscript Kit (Invitrogen). QRT-PCR was performed using a Bio-Rad CFX96 System, with Ssofast EvaGreen Super mix and sequence-specific primers. The melting curve was verified so that a single product was amplified. For quantitative analysis, all samples were normalized to β-actin expression using the ΔΔCT method.

### siRNA

HEK293 and NCI-H295R cells were transfected with ESD RNAi duplex or Klotho RNAi (Supplementary Table S1) for 48 hours using Lipofectamine 2000 (Invitrogen) according to the manufacturer’s instructions.

### Statistical analysis

The data were analyzed by one-way analysis of variance (ANOVA). The unpaired *t*-test was used for comparisons between two groups. Significance was set at a 95% confidence limit.

## SUPPLEMENTARY MATERIALS TABLE


