# Supplementary material for: Regulation of S-formylglutathione hydrolase by the anti-aging gene klotho
Source: Oncotarget. 2017 Jul 8;8(51):88259–75. doi: 10.18632/oncotarget.19111 (PMC5687603; doi:10.18632/oncotarget.19111)
Supplement: Supplementary file 1 [file oncotarget-08-88259-s001.pdf]

Regulation of S-formylglutathione hydrolase by the anti-aging gene klotho

SUPPLEMENTARY MATERIALS

Supplementary Table 1: Oligonucleotide sequences of Skl and FGH siRNA

Skl clone

Skl5	5'-ACCATTGACAACCCCTACGTGG
Skl3	5'-TTTCTAGAGTGAGGAAGCAAGAGGCCGACACTGGGTTTTGTCAAAGGACTTACTTGAACGTAGTTGTCAACAACTCC
F6H5	5'-CTAGAGATTATAAAGATGATGATGATAAAGGCAGCAGCCATCATCATCATCATCACTAAA
F6H3	5'-AGCTTTTAGTGATGATGATGATGATGGCTGCTGCCTTTATCATCATCATCTTTATAATCT

FGH KO siRNA

SiFGH1	5'-GCUGCAAUAUUAAAGGUGAdTdT	5'-UCACCUUUAAUAUUGCAGCdTdT
SiFGH2	5'-CCUUGUGAAAUCCUAUCCAdTdT	5'-UGGAUAGGAUUUCACAAGGdTdT
SiFGH3	5'-CAACCUUUAUUACUGACCAdTdT	5'-UGGUCAGUAAUAAAGGUUGdTdT
